# PO2/TransformON, an ontology for data integration on food, feed, bioproducts and biowaste engineering

**DOI:** 10.1038/s41538-023-00221-2

**Published:** 2023-09-04

**Authors:** Magalie Weber, Patrice Buche, Liliana Ibanescu, Stéphane Dervaux, Hervé Guillemin, Julien Cufi, Michel Visalli, Elisabeth Guichard, Caroline Pénicaud

**Affiliations:** 1 INRAE, UR BIA, 44316 Nantes, France; 2 INRAE, Univ. Montpellier, Institut Agro, UMR IATE, 34060 Montpellier, France; 3 Université Paris-Saclay, INRAE, AgroParisTech, UMR MIA Paris-Saclay, 91120 Palaiseau, France; 4 INRAE, URTAL, 39800 Poligny, France; 5 INRAE, PLASTIC Platform, 91400 Saclay, France; 6 CSGA, CNRS, INRAE, Institut Agro, Université de Bourgogne-Franche Comté, 21000 Dijon, France; 7 INRAE, PROBE research infrastructure, ChemoSens facility, 21000 Dijon, France; 8 Université Paris-Saclay, INRAE, AgroParisTech, UMR SayFood, 91120 Palaiseau, France

**Keywords:** Scientific community and society, Biotechnology

## Abstract

We are witnessing an acceleration of the global drive to converge consumption and production patterns towards a more circular and sustainable approach to the food system. To address the challenge of reconnecting agriculture, environment, food and health, collections of large datasets must be exploited. However, building high-capacity data-sharing networks means unlocking the information silos that are caused by a multiplicity of local data dictionaries. To solve the data harmonization problem, we proposed an ontology on food, feed, bioproducts, and biowastes engineering for data integration in a circular bioeconomy and nexus-oriented approach. This ontology is based on a core model representing a generic process, the Process and Observation Ontology (PO2), which has been specialized to provide the vocabulary necessary to describe any biomass transformation process and to characterize the food, bioproducts, and wastes derived from these processes. Much of this vocabulary comes from transforming authoritative references such as the European food classification system (FoodEx2), the European Waste Catalogue, and other international nomenclatures into a semantic, world wide web consortium (W3C) format that provides system interoperability and software-driven intelligence. We showed the relevance of this new domain ontology PO2/TransformON through several concrete use cases in the fields of process engineering, bio-based composite making, food ecodesign, and relations with consumer’s perception and preferences. Further works will aim to align with other ontologies to create an ontology network for bridging the gap between upstream and downstream processes in the food system.

## Introduction

Food systems are complex and multidimensional systems defined by the Organization for Economic Co-operation and Development (OECD) as the elements and activities related to the production and consumption of food and their effects, including economic, health, and environmental outcomes. More recently, during the Food Systems Summit 2021, the United Nations scientific group (https://www.un.org/en/food-systems-summit/leadership#scientific-group) recalled that “the food system includes the related resources, the inputs, production, transport, processing and manufacturing industries, retailing, and consumption of food as well as its impacts on the environment, health, and society”. There is an accelerating momentum worldwide to bring consumption and production patterns together in a more circular and sustainable approach, known as a bio-based economy or bioeconomy^1^. In addition, the need to develop more integrative approaches to reconnect agriculture, environment, food, and health has been reaffirmed recently^2^. This nexus approach combines the issue of the sustainability of diets with that of the sustainability of agricultural and food production systems, leading to sustainable agri-food systems.

To address such complex questions, the collection of multi-scale characterization data and the extension of analysis methods are major challenges. Data are collected in various projects and are by nature heterogeneous and multidimensional as they cover nutritional, sensory, physicochemical, rheological, microbiological, environmental, and socio-economic aspects. There is thus a need for standardization of these multidimensional and multisource data to facilitate their discoverability and reusability, to assemble them into new datasets allowing new statistical analyses or meta-analyses. Moreover, with the explosion of the volume of data produced in the digital age, there is a need to formalize knowledge to make it “explicit” and “shareable” not only by humans but also by machines and to enable interoperability between information systems and other digital tools.

The FAIR (Findable, Accessible, Interoperable, Reusable) principles provide guidelines for improving the findability, accessibility, interoperability, and reusability of digital resources^3^. These principles focus heavily on the ability of machines to manage data automatically, with minimal human intervention (https://www.go-fair.org/fair-principles/). In this context, the Semantic Web standards and technologies are promising solutions for structuring, linking, and querying data. The Semantic Web is promoted by the World Wide Web Consortium (W3C), an international organization aiming at the development of standards and technologies for making structured data available for processing by machines on the web (https://www.w3.org/standards/semanticweb/). The term “Linked Data” refers to a set of best practices for publishing structured data on the Web (https://www.w3.org/wiki/LinkedData) and building graph-based knowledge representation^4^. Ontologies play a relevant role in some of the FAIR principles, especially with regard to data interoperability and reusability^5^. As a formal representation of knowledge, ontologies provide logical meaning to the data and the possibility to develop machine-readable data format^6^. Some of them are becoming standards promoted either by the W3C or the Open Biological and Biomedical Ontology (OBO) Foundry (https://obofoundry.org/), a community devoted to the development of interoperable ontologies for the biological sciences.

Among the OBO Foundry ontologies, FoodON aims to cover food products and broad food processing steps^7^ providing a lingua franca for representing knowledge about food. This ontology addresses animal and plant food sources, food categories and products, and other descriptive facets coming from LanguaL, a mature and popular food indexing thesaurus (http://langual.org). LanguaL has been used to index numerous food composition databases, including the U.S. Department of Agriculture (USDA) Nutrient Database for Standard Reference (SR) and the European Food Information Resource (EuroFIR) Network of Excellence^8^. However, in 2015, the European Food Safety Agency (EFSA) developed a standardized food classification and description system called FoodEx2 revision 2 (https://www.efsa.europa.eu/en/data/data-standardization). The system is built upon European legislations for pesticide residues, chemical contaminants, food additives, biological monitoring data of zoonoses and zoonotic agents, and microbiological criteria for foodstuffs. FoodEx2 consists of descriptions of a large number of individual food items aggregated into food groups and broader food categories in a hierarchical parent-child relationship called the “master hierarchy”. Moreover, the FoodEx2’s approach is well-suited to our strategy of following a process of transformation, as the classification is based on the identification of the most relevant treatments to create new natures of products from the raw materials and on the creation of specific food groups for the derivatives obtained with these treatments.

In this paper, we present PO2/TransformON, an ontology on food, feed, bioproducts and biowastes engineering for data integration in a circular bioeconomy and nexus-oriented approach. The objective was to build the ontology by re-engineering non-ontological resources such as FoodEx2 and by reusing existing ontologies to ensure backward compatibility with existing datasets^9–16^, and the integration of other bio-based and food systems information thanks to an ontology network. Data stewardship was our main driver.

This new ontology is a specialization of a core model, Process and Observation Ontology (PO2)^17,18^. The PO2 core model was designed to represent a generic process described by a set of steps and experimental observations available for the input and output components of each step of a process. This generic model is already well adapted to transformation or characterization processes but new needs have been identified to cover the targeted domain and answer new questions. Considering that FoodEx2 is an authoritative reference complementary to the Standard Sample Description (SSD2) at the European level and is used by national agencies in European countries for database annotation and data exchange, we decided to base the construction of PO2/TransformON on FoodEx2 for the food and feed hierarchies. In addition, we built a hierarchy for non-food products to cover the newly defined domain. To this end, the legacy vocabulary of FoodEx2 and other valuable non-semantic resources were transformed into linkable data thanks to the fairification process (https://www.go-fair.org/fair-principles/fairification-process/) and the PO2 core model. In the Results section, we present a new version of the PO2 core model and the domain ontology TransformON. In the Discussion section, we illustrate the relevance of this new ontology to get out of information silos through four concrete use cases in the fields of process engineering, bio-based composite making, food design, and consumer preferences.

## Results

### Evolution of the PO2 core model

Typically, domain-specific ontologies represent concepts in a very specific way, which may make them incompatible with other ontologies. To overcome this drawback, one of the possible solutions is to align the concepts of the domain ontologies into a more general representation provided by upper-level ontologies, ontology design patterns, or core models.

**Fig. 1:**
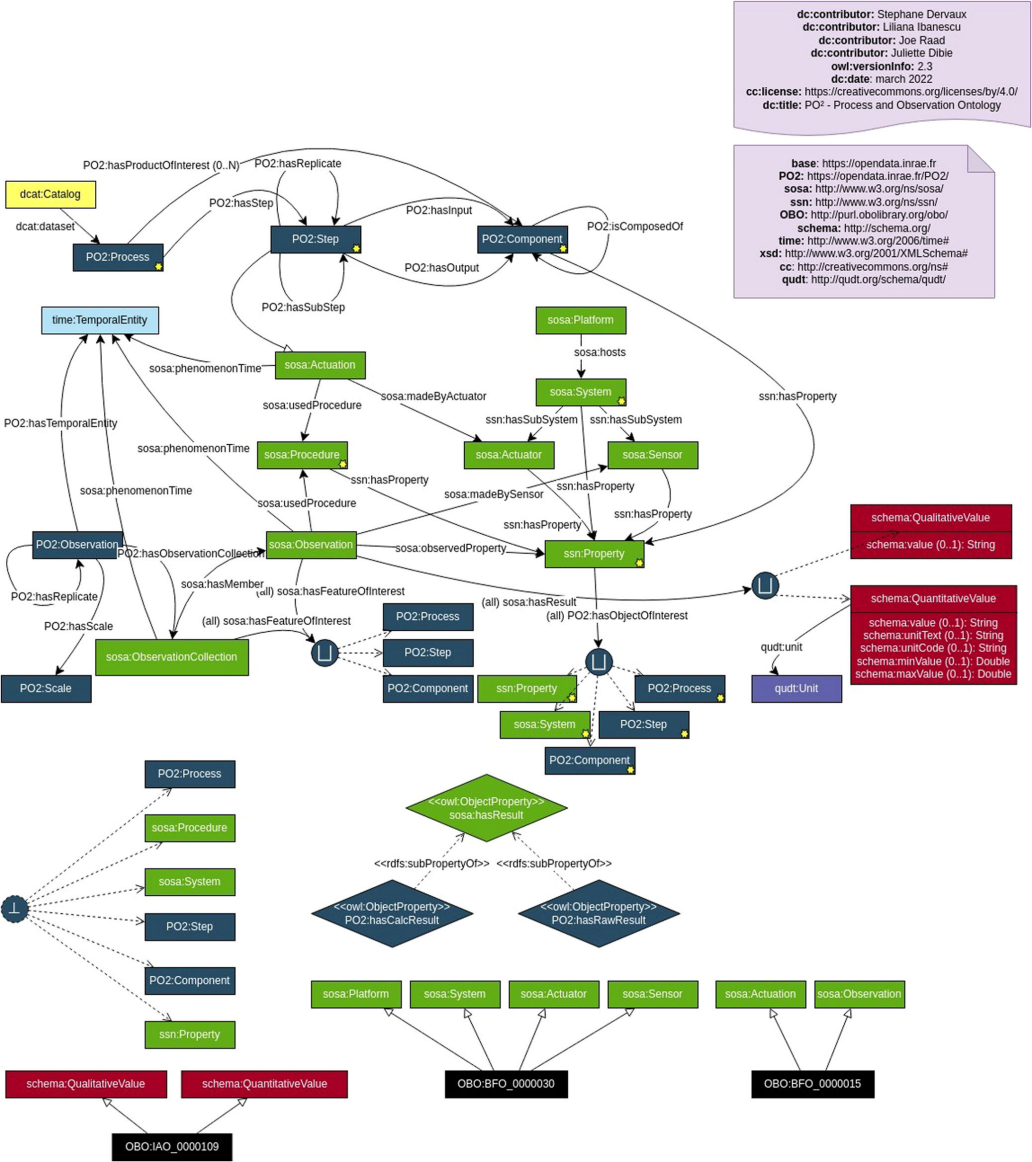
PO2 core model (concepts in dark blue in the upper part: Process part; concepts in green: SOSA Observation part; concepts in red and purple: Result part; concept in light blue: Temporal entity). PO2 concepts correspond to blue rectangles; SOSA/SSN concepts correspond to green rectangles; BFO/IAO concepts correspond to black rectangles; DCAT metadata correspond to yellow rectangles.

The Process and Observation ontology (PO2) is the core model of the domain ontology PO2/TransformON. PO2 is dedicated to the generic modeling of both transformation processes and characterization processes^15,17,18^. PO2 core model reuses various existing ontologies such as SOSA/SSN (https://www.w3.org/TR/vocab-ssn/), Time Ontology (https://www.w3.org/TR/owl-time/), and QUDT (https://qudt.org/) and is also situated within the BFO hierarchy (https://basic-formal-ontology.org/bfo-2020.html). In this section, we present the evolution of the PO2 core model from version V2.2^15^ to V2.3 to meet the new requirements identified during the specification phase of PO2/TransformON (see the Methods section). Figure 1 shows an excerpt of the implemented core model (PO2 V2.3).

The PO2 core model (V2.3) is organized into three parts:

- The Process part that describes the sequence of steps and the products of interest of a process (concepts in dark blue in the upper part in Fig. 1). Each step is linked to a temporal duration and has a collection of input and output components.
- The Observation part that reuses the SOSA/SSN concepts to describe the measurements made (what) and the conditions under which these measurements were obtained (how): concepts in green in Fig. 1.
- The Results part deals with the values obtained from the observation and the units of measure: concepts in red and purple in Fig. 1.

A process can be divided into several routes depending on the control parameters or input compositions considered in the experimental design. Thanks to PO2, the observed properties are linked, on the one hand, to the process, steps, or input/output compositions (sosa:hasFeatureOfInterest) and, on the other hand, to the control parameters or characteristics (ssn:Property) associated with an object of interest during the process (PO2:hasObjectOfInterest). Both quantitative and qualitative variables are described as results of the observation part (sosa:hasResult). PO2 Observation is a core concept that allows the grouping of several sosa:ObservationCollection which represents data tables. The Unified Code for Units of Measure (UCUM, https://ucum.org/) is used for unambiguously representing measurement units. Finally, data are typed according to Schema.org vocabulary (Schema.org, https://schema.org/), and metadata from the Data Catalog vocabulary (DCAT, https://www.w3.org/TR/vocab-dcat-2/) are associated with the dataset corresponding to the process (in yellow in Fig. 1). Datasets are stored in an RDF graph database (also known as a triple store) named PO2 BaGaTel.

The new features of PO2 V2.3 are the addition of two SOSA/SSN concepts (sosa:Platform and sosa:System) to be able to list the equipment of a facility or experimental platform and some additional relations (PO2:hasObjectOfInterest, PO2:has ProductOfInterest, PO2:hasReplicate) to better specify the products of interest in a process or to reduce the ambiguity on the objects that are observed, as well as experimental replications. Thanks to the introduction of PO2:hasObjectOfInterest, PO2 core model is compliant with the InteroperAble Descriptions of Observable Property Terminology (I-ADOPT, https://www.rd-alliance.org/groups/interoperable-descriptions-observable-property-terminology-wg-i-adopt-wg), a general framework for representing the variables derived from observations. Besides, DCAT metadata were added to document the dataset annotated with the ontology.

Seven classes of the core model may be specialized for a specific domain, namely PO2:Process, PO2:Step, PO2:Component, PO2:Scale, PO2:Material (sosa:System), PO2:Method (sosa:Procedure), PO2:Attribute (ssn:Property).

### PO2/TransformON domain ontology

**Fig. 2:**
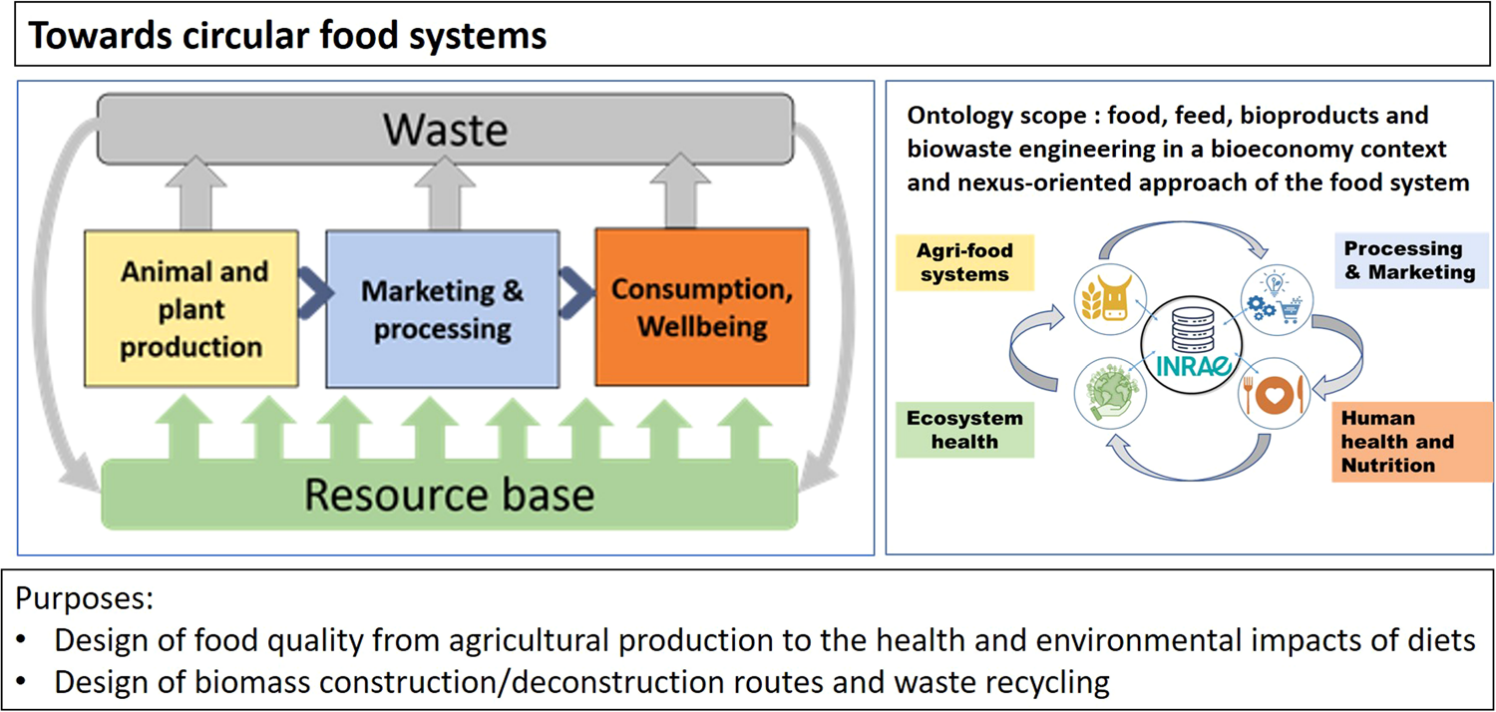
Overview of the scope and purposes of the domain ontology. The domain covers food systems from animal and plant production to the end products, including waste recycling (left-end panel). The scope is given in the right-hand panel and the purposes on the bottom.

The domain ontology PO2/TransformON aims to cover all the aspects related to food, feed bioproducts, and biowastes processing, as well as the characterization of the functional properties of the raw commodities, primary derivatives, end products, and wastes or residues from the treated biomass (Fig. 2). Moreover, the ontology PO2/TransformON aimed to integrate some concepts and classes of other domain ontologies already used for the annotation of existing datasets (DS): DS1) milk microfiltration (10.15454/5MQMKG), DS2) biorefinery (10.15454/X2MOWO), DS3) transmat (10.15454/NK24ID), DS4) PO2 Dairy gels (10.15454/THZ9I1).

**Fig. 3:**
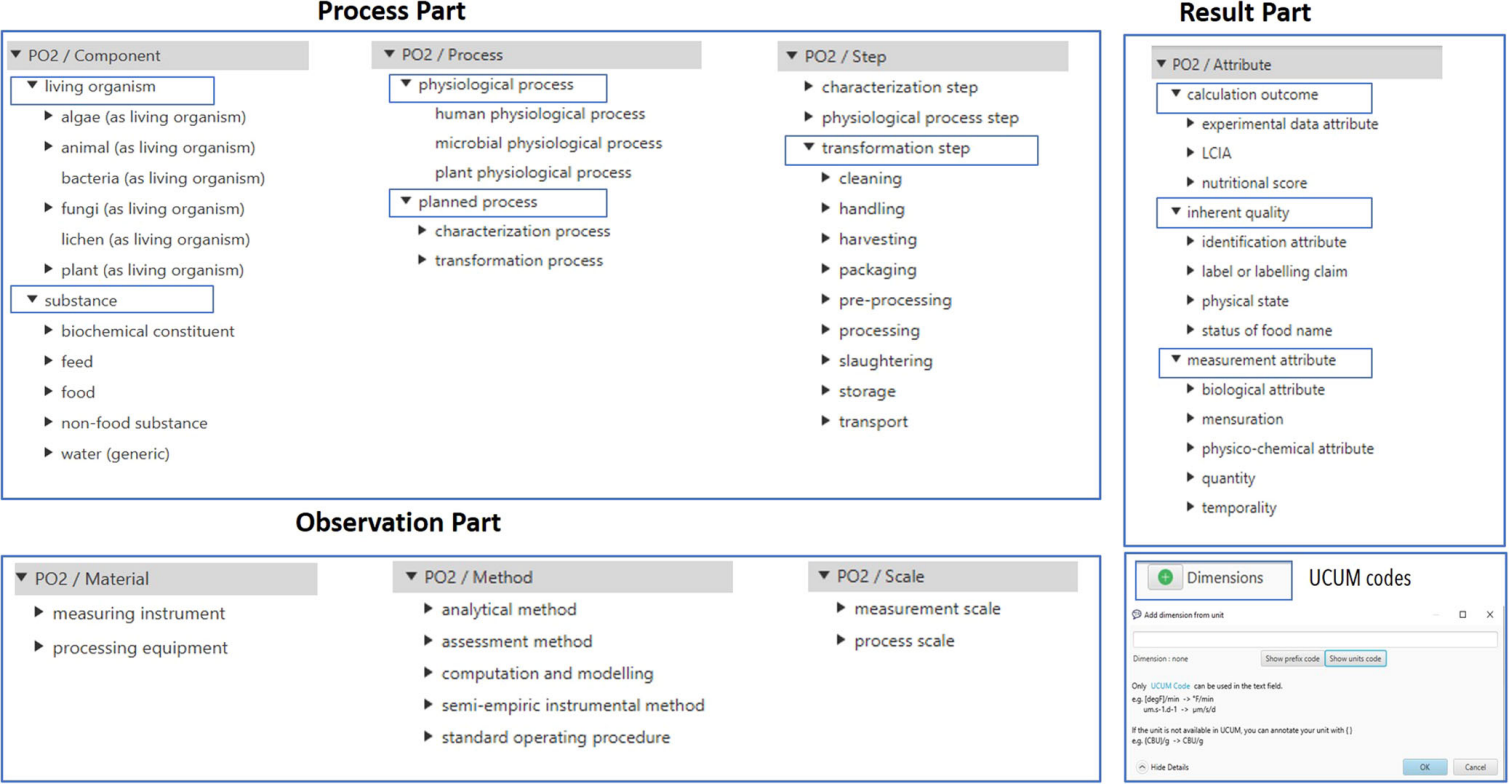
Overview of the three parts of the PO2/TransformON domain ontology (Process part: PO2/Component, PO2/Process, PO2/Step; Observation part: PO2/Material; PO2/Method, PO2/Scale; Result part: PO2/Attribute and UCUM codes). Only the 3 first top levels are shown.

Figure 3 shows the top levels of the PO2/TransformON domain ontology together with the UCUM module allowing the standardization of units of measure.

### PO2/TransformON “Process”part

The “Process” part of the PO2 core model consists of the “Component”, “Process” and “Step” concepts, related to temporal entities. These concepts are specialized as hierarchies of terms covering the studied domains.

#### Component hierarchy

When building the Component hierarchy, first, we have taken into account the need to distinguish living organisms from inert substances (i.e., matter, energy, chemical compounds…). Living organisms are the sources of raw commodities entering the transformation processes. The “Living organism” subclass gathers the categories of living organisms according to the common names of these organisms. It includes the main subclasses covering the natural sources listed in FoodEx2. Subsequently, alignments to taxonomic resources can be made to link these common names to scientific taxa (for example, the National Center for Biotechnology Information (NCBI) taxonomy, https://www.ncbi.nlm.nih.gov/taxonomy). The “Substance” subclass allows us to classify the substances into five subsequent categories: food, feed, non-food substances, biochemical constituents, and water (generic).

**Fig. 4:**
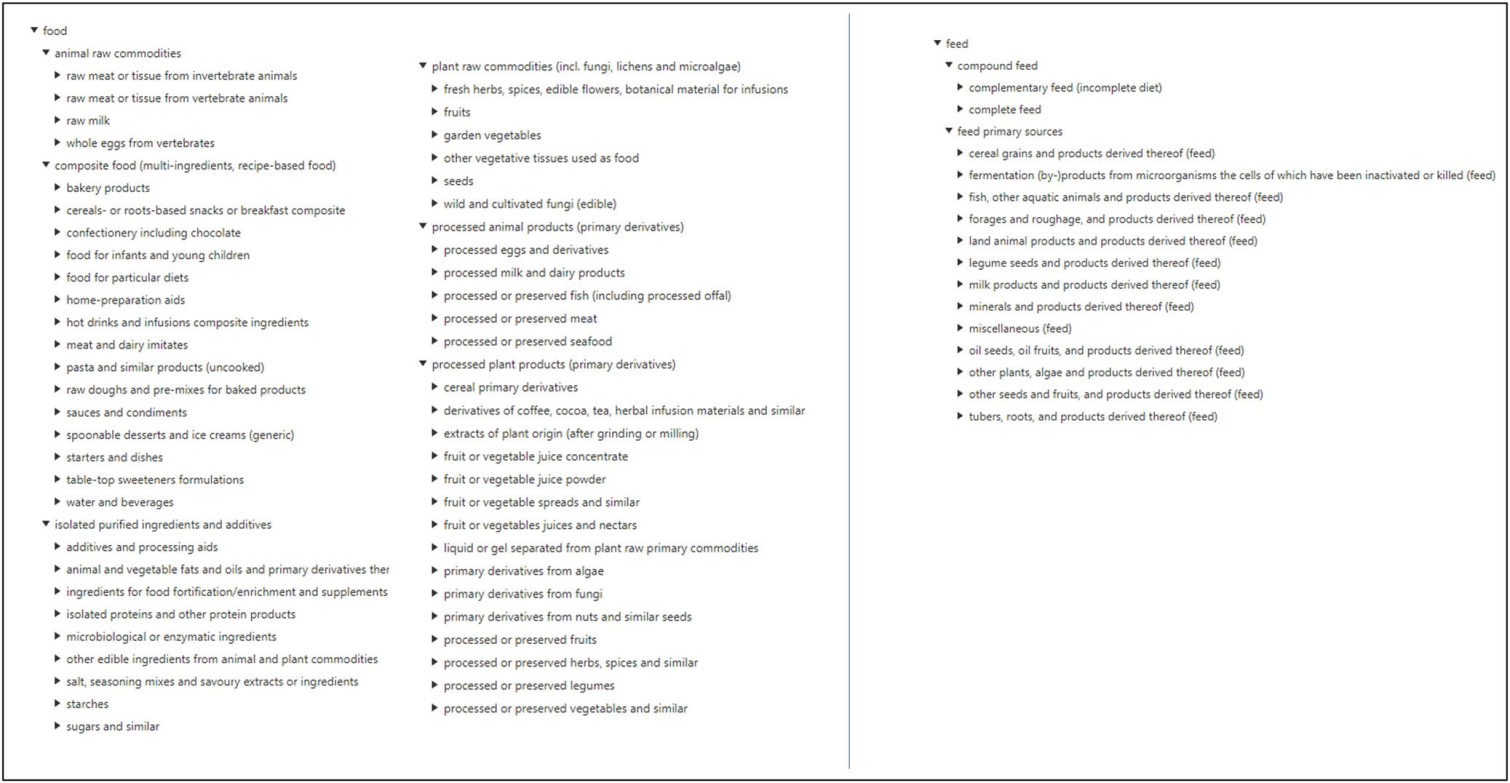
Food and Feed hierarchies (only the first 3 top levels are shown). Food hierarchy is shown in the first two columns. Feed hierarchy is shown in the third column.

The novelty and the contribution of our work lie in the building of a hierarchy allowing the integration of the food, feed, and non-food part: on the one hand, we have made up classes from the “Food” and “Feed” hierarchies proposed in the FoodEx2 frame of reference, and on the other hand, we have created classes for “Non-food substances”, i.e., chemical compounds or residual organic substances which are used, produced or emitted during transformation processes. Figure 4 shows the Food and Feed hierarchies.

#### Food classes

The “Food” branch is based on the master hierarchy proposed in FoodEx2. It includes 4234 classes directly imported from FoodEx2. The “Food” branch is divided into five main subclasses with respect to the source of raw commodities (animal or plant origins) and the degree of transformation (raw, derivative, composite food, and isolated purified ingredients). The food components from the datasets milk microfiltration (DS1) and dairy gels (DS4) were added to this hierarchy.

#### Feed classes

**Fig. 5:**
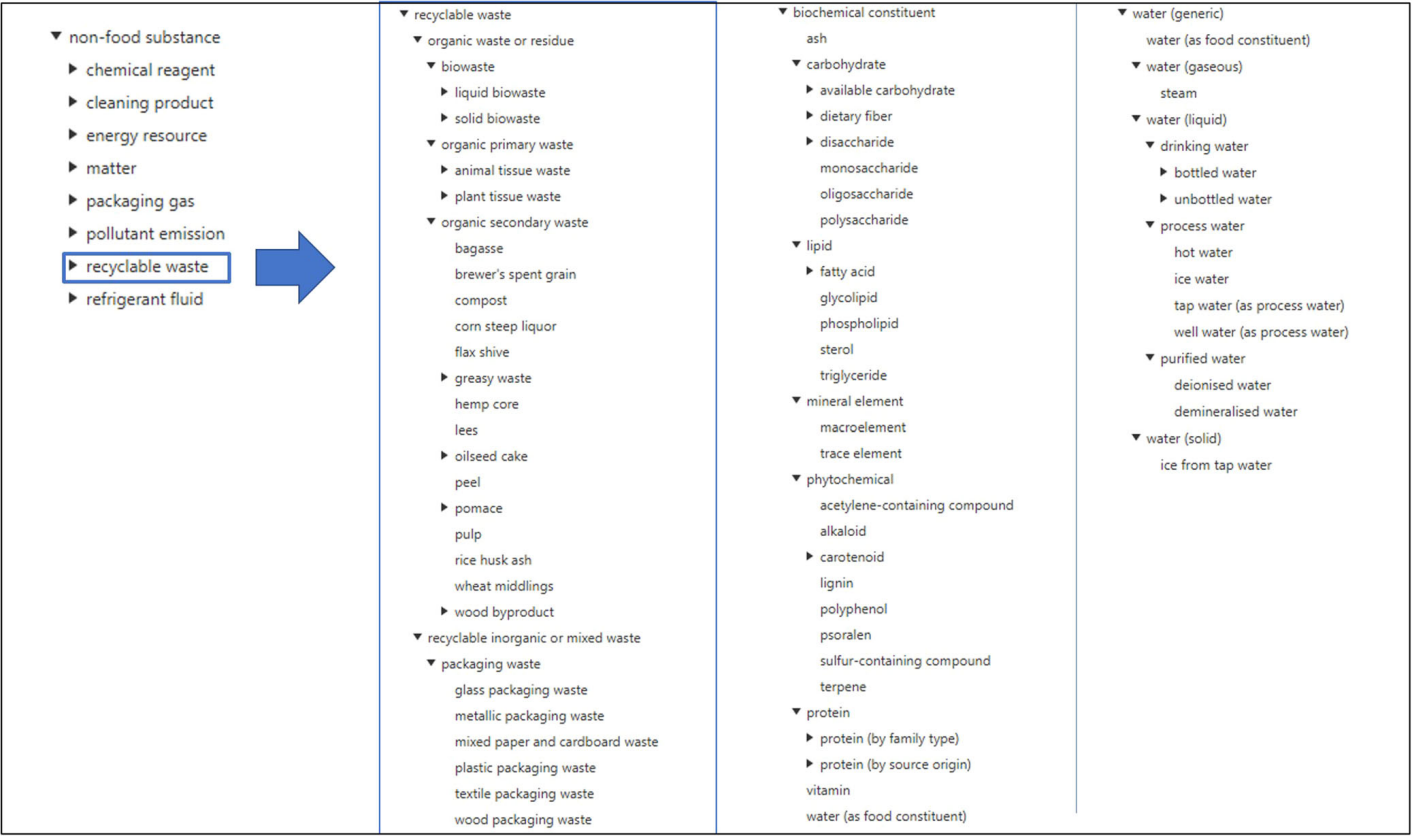
Non-food substance, biochemical constituents, and water class hierarchies, with a focus on recyclable waste sub-hierarchy. Only the first top levels are shown. The non-food substance hierarchy is shown in the first column. The blue arrow shows the recyclable waste details. Biochemical constituents and water class hierarchies are shown in the third and fourth columns, respectively.

The “Feed” branch includes 759 classes imported from FoodEx2. However, two main subclasses were created: one for grouping all the primary sources of feed and the other one for grouping the compound feed. Figure 5 shows the non-food substances, biochemical constituents, and water class hierarchies, with a focus on recyclable waste.

#### Non-food substance classes

The “Non-food substance” branch was created in PO2/TransformON to group all the chemical substances used or produced during transformation processes, taking into account the role or nature of the substances, namely whether they are organic or inorganic: chemical reagents, cleaning products, energy resources, matter, packaging material, packaging gas, refrigerant fluids, polluting emissions, and recyclable wastes.

The construction of the “recyclable waste” branch of PO2 was based on existing reference systems such as the European Waste Catalogue (EWC), a hierarchical list of waste descriptions established by the European Commission decision 2000/532/EC2 to harmonize the different nomenclatures existing in the Member States. The European Waste Catalogue defined 20 main categories with respect to the origin of waste and further disposal or recycling treatment. For classifying the wastes and residues from biomass, we first took into account the “non-hazardous organic waste” categories such as “wastes from agriculture, horticulture, aquaculture, forestry, hunting and fishing, food preparation and processing” and “wastes from wood processing and the production of panels and furniture, pulp, paper, and cardboard”. We then defined a “recyclable waste” class and further divided it into “recyclable organic waste or residue” and “recyclable inorganic or mixed waste” to include packaging wastes. Lastly, following the same logic as for the “Food” and “Feed” branches of FoodEx2, namely the degree of transformation, we created classes of “primary organic waste” (animal or plant tissue waste or residues from agricultural production), “secondary organic waste” (residues or by-products from transformation processes), and “residual biowaste” (liquid or solid organic final wastes, including sludges and liquid wastes from waste treatment). The concepts used in the biorefinery and transmat datasets (DS2 and DS3) were also integrated into this hierarchy.

The “non-food substances” hierarchy includes 270 classes which can be further specialized into additional subclasses for other types of by-products and waste encountered in future use cases. Concepts that refer to chemical substances will be aligned with CheBI (https://www.ebi.ac.uk/chebi/init.do) or PubChem database (https://pubchem.ncbi.nlm.nih.gov/).

#### Biochemical constituents and water classes

Finally, the “Substance” hierarchy includes two other subclasses for biochemical constituents and different forms of water. The “biochemical constituents” subclass groups all the components that are part of food or feed products (i.e., nutritional compounds or dietary compounds). The “water (generic)” subclass allows us to group the different types of water that can be encountered in different physical states or forms such as drinking water, process water, purified water, or water found as a food constituent. The concept “water (as food constituent)” belongs to both subclasses of generic water and biochemical constituents in a parent-child relationship thanks to polyhierarchy.

The biochemical constituents and water (generic) branches currently include 61 and 29 main classes respectively, but mappings to OBO Foundry ontologies such as FoodON, CDNO, and CheBI will provide other specific concepts dealing with dietary or chemical constituents^7,19^.

### Process and step hierarchies

**Fig. 6:**
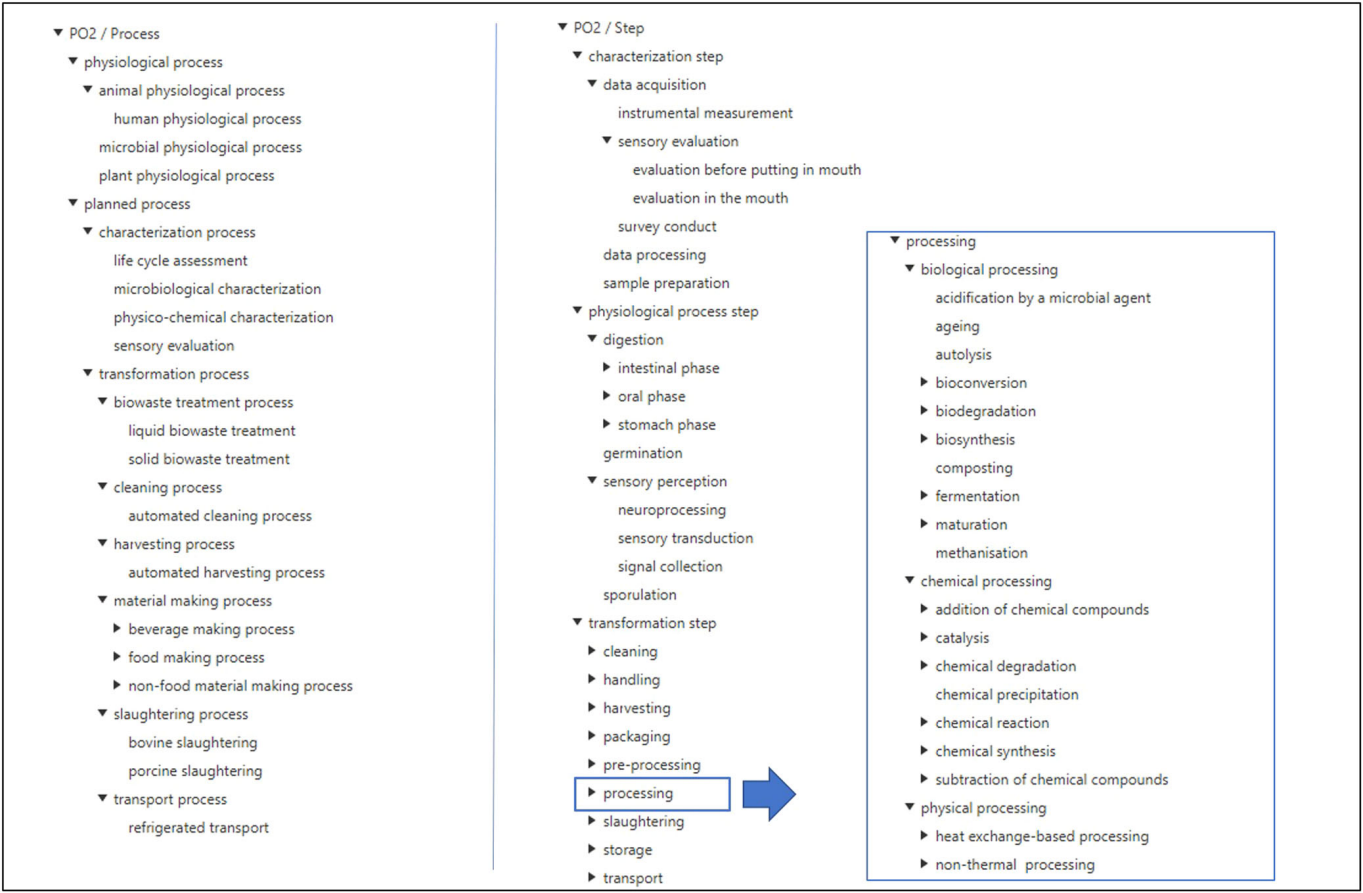
Process and step hierarchies. Only the first top levels are shown with a focus on further levels for processing steps.

Figure 6 shows the process and step hierarchies with a focus on processing steps.

#### Process classes

Two process types have been defined in the PO2/TransformON ontology. Planned processes are processes that follow procedures, whereas biological or physiological processes are unplanned processes. TransformON covers both planned processes for food and non-food transformation or characterization and includes 33 classes. The hierarchy also covers unplanned processes such as spontaneous fermentations or human digestion which will be extended in an upcoming work with the FoodON curation group^7^ and OBO Foundry community. Mappings to OBI (https://obi-ontology.org/) or COB (https://obofoundry.org/ontology/cob.html) will thus provide a connection to analytical protocols and biological processes.

#### Step classes

Steps are the elementary entities that compose a process itinerary. The step sub-hierarchy includes 396 classes which were grouped according to the kind of process they belong to (i.e., physiological steps, characterization, or transformation steps). These main subclasses were further divided into subsequent subclasses specializing in steps according to the type of event or action they represent. It is worth mentioning that the transformation steps were further divided into several levels of subclasses according to the different nature of the operation (biological, chemical, and physical) involved. The list of steps was taken from the FoodEx2 process hierarchy, internal communications, and books^20,21^. The unit operations encountered in the four use cases were also included.

### PO2/TransformON “Observation” and “Result” parts

**Fig. 7:**
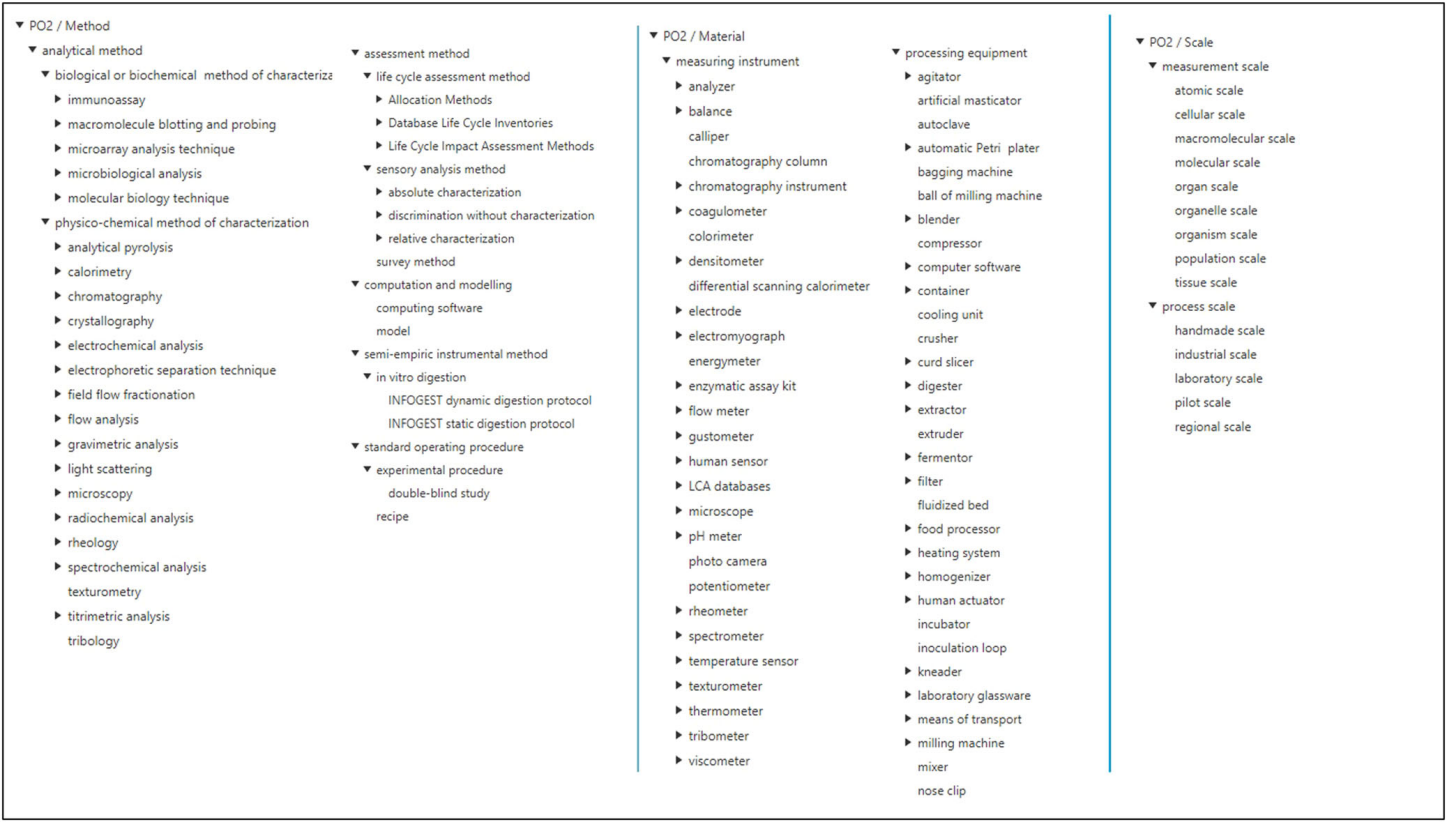
Material, method, and scale hierarchies. Only the first top levels are shown.

The observation part of PO2/TransformON includes the core concepts that enable the description of materials, methods, and scale of the observation. The observable properties are specialized in the PO2/TransformON attribute hierarchy. Figure 7 shows the material, method, scale et attribute hierarchies.

- PO2 Material: this concept represents systems in the sense of SOSA/SSN, which can be constituted of either a transformation equipment (sosa:Actuator), or a measuring instrument (sosa:Sensor). Actuators and sensors can be devices or human agents (e.g., a tasting panel).
- PO2 Method: this concept represents procedures in the sense of SOSA/SSN: they can be either techniques and analytical protocols, or operating instructions and recipes.
- PO2 Scale: this concept allows specification of the size of the transformation process (process scale) and/or the size of the observed object (measurement scale).
- PO2 Attribute: this concept represents observable properties in the sense of SOSA/SSN. An observable property is any characteristic that describes an object of interest, i.e., a component, a material, a method, an attribute, a step or a process.

The attributes were grouped into three main subclasses: calculation outcomes (characteristics whose values are calculated from other values), intrinsic qualities (characteristics inherent to the objects), and measurement attributes (characteristics obtained from measurement). Moreover, some specific attributes, materials, and methods were included for Life Cycle Assessment (LCA) purposes. For example, the life cycle impact assessment (LCIA) subclasses of the PO2 Attributes hierarchy contain the environmental and human health impacts resulting from the elementary flows evaluated with an LCA method^22^.

### Overview of PO2/TransformON building and datasets stewardship

**Fig. 8:**
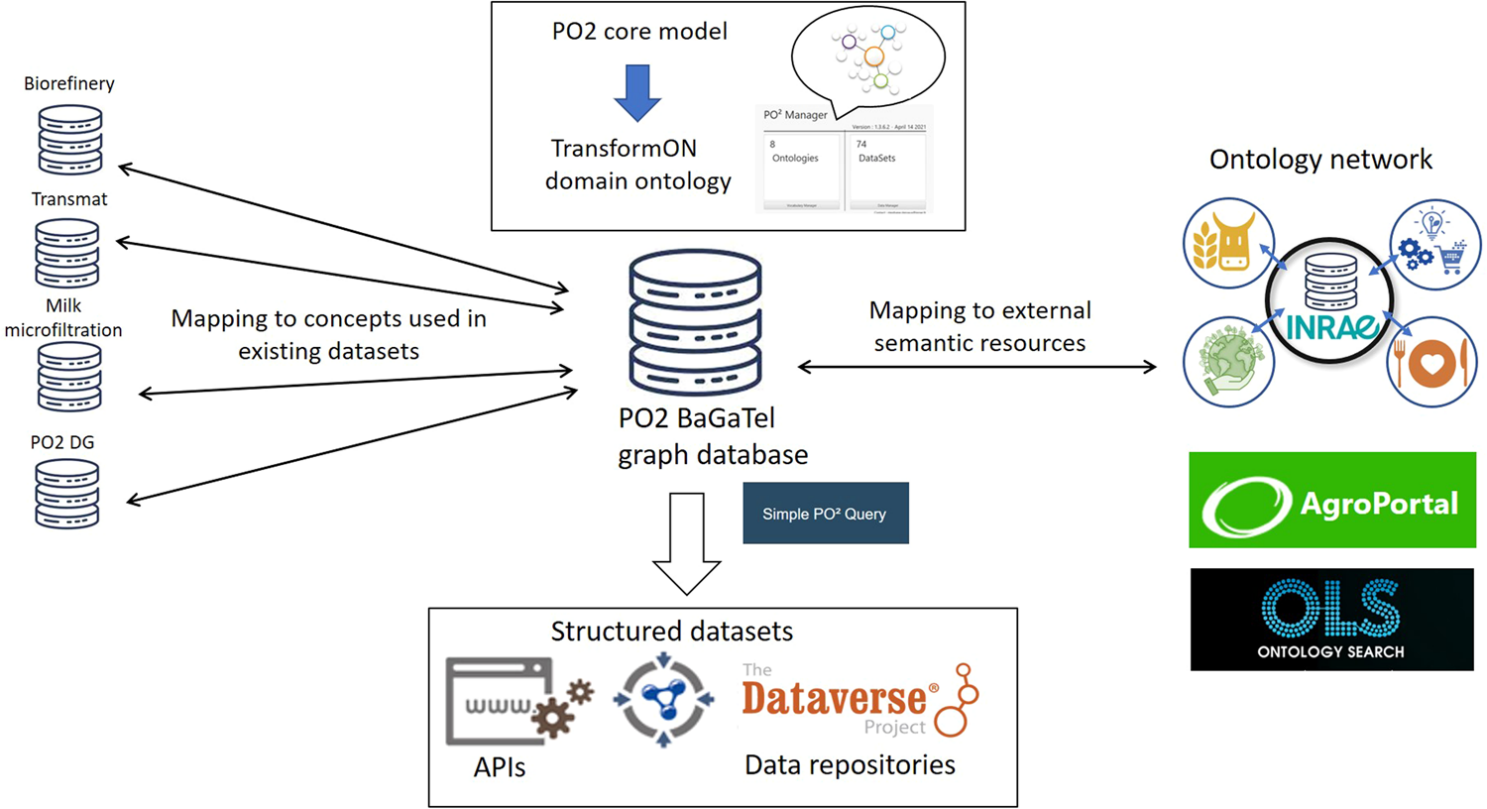
General workflow and the ecosystem of tools for ontology building and data stewardship. APIs Application Programming Interfaces, RDF Resource Description Framework, PO2 process and observation ontology, OLS Ontology Lookup Service.

To conclude this section, it should be emphasized that the PO2 core model and the TransformON domain ontology vocabulary make it possible to structure and annotate the data collected both on the input/output products obtained from biomass and associated processes and steps. This structuring provides specific metadata that makes querying the data more efficient, which is a valuable asset for answering complex questions and breaking out information silos. Figure 8 illustrates the general workflow and the ecosystem of tools for ontology building and data stewardship (see the Method section for more information).

## Discussion

### Evaluation of the reusability and relevance of the domain ontology PO2/TransformON

The objective of this work was to build a new domain ontology by re-engineering non-ontological resources such as FoodEx2 and by reusing existing ontologies to ensure backward compatibility with existing datasets. Beyond the FAIR principles (Findability, Accessibility, Interoperability, and Reusability), the evaluation of the ontology should also take into account its relevance to address specific use cases and applications.

**Fig. 9:**
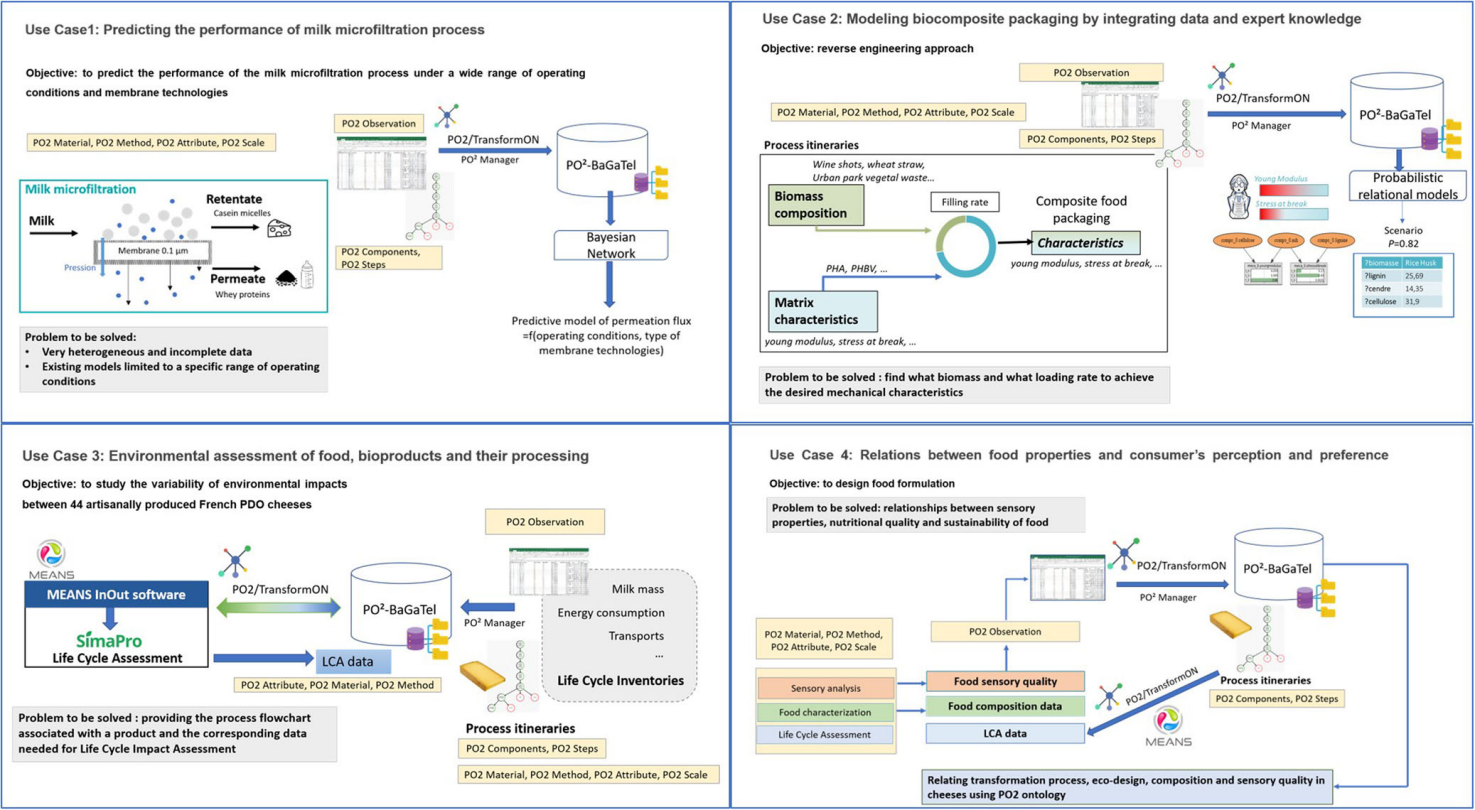
Overview of the four use cases and contribution of PO2 Manager and the domain ontology PO2/TransformON to integrate the data. The four use cases are dealing with process engineering (use case 1), bio-based composite making (use case 2), food ecodesign (use case 3) and relations with consumer’s perception and preference (use case 4). The PO2 concepts are shown in yellow rectangles.

In this part, we will show the relevance of this new domain ontology PO2/TransformON through several concrete use cases in the fields of process engineering (use case 1), bio-based composite making (use case 2), food ecodesign (use case 3) and relations with consumer’s perception and preference (use case 4). We chose these use cases to illustrate each of the sub-domains of ontology in a circular bioeconomy and nexus-oriented approach. The use cases have been previously published in data papers or articles^9–16,23,24^. The fourth use case is still in progress, but it provides a good example of how heterogeneous sources can be integrated and made interoperable thanks to the new domain ontology, which combines the vocabulary previously used in the other use cases. Figure 9 provides an overview of the different use cases and the contribution of the different tools to integrate the data thanks to the new domain ontology PO2/TransformON. All use cases are using the Process, Observation, and Result parts previously described. The use cases 1 and 2 are more specifically focused on reusing the data into probabilistic models, while the use cases 3 and 4 are illustrating how to combine data of different characteristics (organoleptic, nutritional, or environmental aspects).

### Use Case1: Predicting the performance of the milk microfiltration process

Milk microfiltration is becoming more and more attractive in the dairy sector. Cross-flow microfiltration with a 0.1 µm membrane is widely used to separate native casein micelles (~150 nm) from whey proteins (~2–15 nm) in skimmed milk. The concentrated casein retentate is used to standardize cheese milk. The permeate, which contains whey proteins, is ultrafiltered to provide protein-rich concentrates with high nutritional and functional properties. The growing interest in these milk protein fractions explains the development of milk microfiltration equipment. However, this process remains difficult to understand as a whole, in particular, because the existing models^25^ only evaluate a precise range of operating conditions and do not take into account the different types of membrane technologies that can be used^26^. To meet this challenge, Baudrit et al.^27^ designed a method combining the use of the PO2/TransformON vocabulary and a Bayesian network, to structure heterogeneous data sources, assess the reliability of data sources, and provide relevant recommendations based on deductive and quantitative reasoning. This innovative method allows answering specific questions from experts in the field, namely to predict the performance of the milk microfiltration process under a wide range of operating conditions and membrane technologies.

### Use Case 2: Modeling biocomposite packaging by integrating data and expert knowledge

The massive amount of plastics used each year is causing waste to accumulate in our environment. Faced with the depletion of fossil fuels and the increase in organic residues (e.g., agricultural, urban, forestry), the development of new technologies to create bio-based and biodegradable composite materials makes it possible to provide a recovery solution while producing replacements for plastic. Tailoring the design of biocomposite materials increases the need to understand and model the links between the structure of the materials and their final performance (e.g., water vapor permeability, thermal and mechanical characteristics). To this end, Münch et al.^15^ designed and implemented a digital workflow for transformation processes, called POND (Process and observation ONtology Discovery) to support reasoning under uncertain conditions based on experimental data and expert knowledge. This workflow allows predictions to be made by a learning system based on two models: i) PO2 core model and ii) probabilistic relational models (PRMs). Combining them in POND allows users to perform a retroactive operation, where each learned model is submitted to the expert who can refine the integrated knowledge or add new knowledge. When applied to a dataset from five different projects, POND has proven to be effective in enabling the formulation of optimal biomass types based on the desired characteristics of the final product, and finding another potentially interesting biomass even if it was not initially tested in the five projects^16^.

### Use Case 3: Environmental assessment of food, bioproducts, and their processing

There is an urgent need to reduce the environmental damage caused by food systems^28^, all the more so the world’s population is expected to grow to 9.7 billion by 2050^29^, which will increase the need for food production. Widely applied to the agri-food sector^30^, Life Cycle Assessment (LCA) is the most scientifically recognized environmental analysis method worldwide and is standardized^31^. It allows quantifying the environmental impacts of a product, process, or service during its whole life cycle. However, LCA is a data-intensive method which is often a crucial limit in the quality and quantity of LCA^32^. The PO2 BaGaTel RDF graph database is helpful in providing the process flowchart associated with a product and the corresponding data needed for LCA^23^. PO2 core model and TransformON vocabulary are key-elements for retrieving available data: when the exact query could not be found, the hierarchy levels may be explored in order to find alternative data from different projects. For instance, if data on ‘Comté cheese’ cannot specifically be found, a query can be done on the upper level ‘Cheese’. In the same way, with regards to processing, if the electrical consumption of the ripening step cannot directly be found, it can be calculated from the existing data on the equipment (here from the power of the refrigerating system and the duration of the ripening). Complementary, PO2/TransformON has supported the development of the food and bioproduct engineering part of MEANS InOut tool, a user-friendly web app that helps users to perform LCA^22^. The usefulness of TransformON lies in the structured description of ingredients (inputs), products (outputs), processes, and steps, assisting LCA practitioners to describe their production systems through drop-down menus. A further step could be to align PO2/TransformON with open LCA databases, as well as food composition databases, in order to perform a multicriteria assessment of food.

### Use Case 4: Relations between food properties and consumer’s perception and preference

Food choices are difficult to change, and consumers’ motivations can be incompatible with the food transition^33^. If the reformulations of food products tend towards environmental and nutritional benefits, ensuring consumer adherence to the food transition requires that the reformulated products remain acceptable. Indeed, the taste is the main reason for food purchases in many countries^34–38^. Thus, investigating the perception of food products by consumers is crucial for the comprehension of the determinants that drive their acceptance and eating behavior. This can be achieved by capitalizing on existing data obtained from published studies involving different sensory evaluation methods. However, this approach requires a typology of the sensory evaluation measures and descriptors in order to define when and how data can be aggregated. A first proposal for a data-centric typology of sensory evaluation measures^39^ has been included in the Method’s hierarchy of TransformON. Work is also underway to ensure that the Attribute’s hierarchy of TransformON incorporates as many sensory descriptors as possible. Bondu et al. proposed a lexicon and a generic wheel of texture descriptors^40^ and work is under progress to include the aroma/odor, flavor, and trigeminal descriptors. The Component’s hierarchy of PO2/TransformON conforms to the FoodEx2 classification and is a central reference point for food description and mapping with FoodON. Going further will require aligning PO2/TransformON with other food and consumer-related data sources, such as food composition databases, or epidemiological survey databases. The development of new methods adapted to massively collect data relating to consumer perception or preference is also underway. Extracting relevant sensory information from textual sensory descriptions expressed with consumers’ own vocabulary requires combining semantics with Natural Language Processing tools. The hierarchical classification of sensory descriptors proposed in PO2/TransformON will serve as a basis for the semantics.

To conclude, the PO2/TransformON domain ontology provides a vocabulary designed for specific needs to describe transformation processes and characterize food, bioproducts, and biomass waste. Based on the experience acquired after the use cases study and implementation, the main strengths and limitations of the approach are the following: this work makes it possible to structure heterogeneous data and expertize from different domains in a homogenized structure given by the core ontology PO2 that allows reasoning. Moreover, sharing a common reference system enables us to uniformly query available data and to break out of the information silos that usually constitute individual projects. The approach permits contextualizing experimental data with metadata about material and method in order to be able to either redo experimentations or decide if data acquired in different research projects are comparable for meta-analysis perspectives. This also allows to identify existing knowledge gaps and suggest new experiments to be conducted. When coupled with Bayesian networks, the system can be used for learning and prediction under uncertainty. The ecosystem of tools was designed to assist domain users in building the vocabulary, collecting, querying, and publishing data using Semantic Web technologies, but the price to pay remains high as it takes effort to understand the PO2 model, and manually collecting data is a time-consuming and error-prone task. We are therefore still working on the development of an automated import template. Finally, the developed software tools enable the publication of data structured in the PO2 RDF format on the INRAE’s open data repository hosted on the Research Data Gouv platform (https://entrepot.recherche.data.gouv.fr/dataverse/inrae) for long term preservation and sharing.

The main outcomes of the work presented in this paper consist in the evolution of the PO2 core model, the semantization of FoodEx2, the re-engineering of existing domain ontologies, and the creation of a reference system providing unique resource identifiers (URIs) for food, bioproducts, and biowastes engineering. In addition, the integration of sensory aspects in the vocabulary constitutes an added value compared to the purely “process” and “safety” points of view initially taken into account in FoodEx2. Ultimately, we also plan to align PO2/TransformON to other ontologies to create an ontology network for bridging the gap between upstream and downstream processes in the food system.

## Methods

### Knowledge management in the context of semantic web technologies

Ontologies seek to formally describe a domain of knowledge by identifying the objects in this domain, their properties, and their relationships. For ontologies representation in the context of the Semantic Web, the OWL2 Web Ontology Language (https://www.w3.org/OWL/) is standard. However, the Simple Knowledge Organization System (SKOS, https://www.w3.org/TR/skos-reference/) is a less formal data model that allows representing thesauri, classifications, or other types of controlled vocabularies. In SKOS, conceptual resources (concepts) are identified with unique resource identifiers (URIs), labeled with strings in one or more natural languages (multilingual terms), documented with various types of notes, semantically related to each other in informal hierarchies and association networks, and aggregated into concept schemes. Using SKOS, more human-readable information (labels and documentation) may be added to an existing formal ontology. Therefore, we used SKOS to create the hierarchies of concepts that we defined as OWL classes in the ontology.

OWL2 and SKOS use the Resource Description Framework (RDF, https://www.w3.org/RDF/) data model that is readable and interpretable by machines. RDF extends the linking structure of the Web by using URIs to name the relationship between things as well as the two ends of the link, the subject and the object (this is usually referred to as a “triple”). Using this simple model, it allows structured and semi-structured data to be mixed, exposed, and shared across different applications. This linking structure forms a directed, labeled graph, where the edges represent the named link between two resources, represented by the graph nodes. Sparql Protocol and RDF Query Language (SPARQL) (https://www.w3.org/TR/rdf-sparql-query/) is the query language for RDF recommended by the W3C.

### Overview of the methodology used for ontology engineering

To design the PO2/TransformOn ontology, we followed the Linked Open Terms (LOT) Methodology, a reuse-based lightweight method for developing Linked Data ontologies and vocabularies^41^. This methodology aims to be compatible with software development techniques in which sprints and iterations represent the main workflow organization in order to align ontology development with software development agile practices. In addition, the methodology focuses on: (a) the reuse of terms (ontology classes, properties, and attributes) existing in already published vocabularies or ontologies and (b) on the publication of the built ontology according to Linked Data principles. It is also worth mentioning that the LOT methodology builds on top of the ontological engineering activities defined in the NeOn methodology^42^, and the KNowledge Acquisition and Representation Methodology (KNARM)^43^.

The LOT methodology (https://lot.linkeddata.es/) defines iterations over a basic workflow composed of the following activities: (1) Ontological requirements specification; (2) Ontology implementation; (3) Ontology publication; and (4) Ontology maintenance

However, as we reused the PO2 core model v2.2 previously described^15,44^, we did not proceed from scratch. Most of the work, therefore, consisted in analyzing existing concepts and terms, collecting and reorganizing them by adding new terms to complete the vocabulary. In that way, we specialized the PO2 core model to cover the newly defined domain.

The next section details the procedure followed for the building of the domain ontology TransformON based on the PO2 core model. Our procedure adapted from LOT is divided into three activities. The first activity defines the ontology requirements associated with the desired outcome, namely the PO2/TransformOn domain ontology. The second activity represents the selection of semantic and non-semantic resources and the gathering of the required concepts for ontology formalization and conceptualization. The third activity describes the ontological transformations used to integrate the selected resources into PO2/TransformOn in order to fulfill the requirements. The publication and maintenance strategy of the ontology is detailed in the section presenting the ecosytem of tools.

### Procedure for PO2/TransformON ontology creation

PO2/TransformON ontology creation was done in three steps: (1) specification step, which consisted in identifying the scope and the objectives, competence issues, and relevant use cases; (2) formalization and conceptualization step, which consisted in identifying the most generic concepts and distinguishing the most specific concepts, in order to establish the hierarchy of specialization of the PO2 core model; and (3) implementation, publication and maintenance step, which consisted in encoding, providing URIs and mapping to external semantic resources or the domain ontologies previously used for the annotation of the datasets.

### PO2/TransformOn requirements specification

The ontology requirements specification activity refers to collecting the requirements that the ontology should fulfill. The ontology development team worked in collaboration with users and domain experts to define the purpose and scope of the ontology to be developed. Communication between domain experts, users, and the ontology development team occurred through monthly online and physical meetings between January 2021 and June 2022. Experts were engineers and researchers working in INRAE (France’s National Research Institute for Agriculture, Food and Environment, https://www.inrae.fr/en).

First, we defined the purpose and scope of the new domain ontology aimed to structure and build a federated graph-oriented database thanks to a harmonized vocabulary aligned with international reference systems, to allow knowledge integration and further modeling and reasoning. The objective of the domain ontology is to cover the whole field of Food and Bioproducts engineering, including transformation from raw materials to end-products and the characterization of the quality and usage properties of products, including by-products and waste. Within this scope, four sub-domains were identified: (i) characterization of foodstuffs and food ingredients (ii) characterization of bioproducts (including water and recyclable waste), (iii) food and non-food processing (physical, chemical, and biological planned processes) and life cycle assessment (LCA), and (iv) sensory perception, oral physiology and digestion processes, related to consumer preferences, nutrition and health (e.g., food allergenicity, nutrient bioavailability, etc). Four use cases were then selected, one in each identified sub-domain, as a source of information for defining the specific vocabulary to be included in the domain ontology.

During this requirements specification phase, new ontology requirements were identified, which implied considering some evolutions of the PO2 core model. The newly defined requirements were the following:

- to model a global process of biomass transformation by being able to distinguish between food and non-food products (OR1),
- to be able to distinguish between what comes from primary production, secondary processing, and waste (OR 2),
- to represent the experimental observations throughout the process by being able to distinguish the object of interest in the observation (OR3),
- to represent the list of equipments of a given platform (OR4),
- to be able to retrieve the replications of a process with respect to an experimental design (OR5)
- to identify metadata allowing traceability and harvesting of the ontology and corresponding datasets once published on the Web (OR6)

A list of competency questions (CQs) was also collected. Competency questions are natural-language questions that outline the scope of knowledge represented by an ontology^45^. These CQs allow identifying basic concepts and the relationships between those concepts.

Typical CQs addressed by the domain ontology covering the field of Food and Bioproducts engineering are for example:

- CQ1: Which steps compose a given transformation process?
- CQ2: Which material is involved in the process?
- CQ3: Which attribute values are associated with each step?
- CQ4: What are the attribute values associated with an input (or output) for a given step of a given transformation process?
- CQ5: What are the changes for an attribute value of an input (or output) during a given step?
- CQ6: Which steps of the process were replicated?

CQs for one specific use case, for example, pizza making, can be expressed as follows:

- What are the different steps required to produce a pizza?
- What equipment is used to produce a pizza? At what stage(s) is each piece of equipment used?
- What is the quantity of each ingredient being processed in the recipe? What is the unit of quantity?
- What are the average values taken by the sensory attributes for a given level of a nutritional attribute (fat, carbohydrate, protein, etc.)?
- For what lipid content is the salty intensity note maximum?
- How much water is needed to clean the equipment?
- What is the consumer preference score for each pizza?

As the output of this first activity, a document was produced describing the purpose and scope of the ontology, as well as the ontology requirements and CQs.

### Collection of terms and concepts for ontology formalization and conceptualization

To collect the terms and concepts required for knowledge representation in PO2/TransformON, we combined two complementary approaches: first, we followed a bottom-up approach with the existing use cases and datasets (data-driven approach), and second, we followed a top-down approach for selecting knowledge resources. From the list of extracted terms and the CQs collected during the specification phase, we searched for appropriate existing resources. This activity consisted in looking for existing ontologies or thesauri that best fit the previously extracted terms (and their synonyms). We used specific services for ontology retrieval such as the Ontology Lookup Service (OLS, https://www.ebi.ac.uk/ols/index), a repository for biomedical ontologies, and AgroPortal (https://agroportal.lirmm.fr/), an ontology repository for agronomy and related domains. We selected FoodON as a main resource with respect to food, as well as other OBO Foundry ontologies (https://obofoundry.org/), namely CheBi, CDNO, OBI, CHMO, PATO, or ENVO, among others. Another major semantic resource selected was the AGROVOC thesaurus (https://www.fao.org/agrovoc/about), a multilingual and controlled vocabulary designed to cover concepts and terminology coordinated by the Food and Agriculture Organization of the United Nations (FAO).

With respect to the scope and purposes defined in the specification phase, we also considered other non-semantic resources, such as nomenclatures and other classification systems. Considering that FoodEx2 is an authoritative reference at the European level, we decided to base the construction of PO2/TransformON on FoodEx2 for food and feed hierarchies and for some parts of the process and step hierarchies. We also took into account the European Waste Catalogue (EWC), a hierarchical list of waste descriptions established by Commission decision 2000/532/EC2 for the non-food hierarchy. The International Union of Pure and Applied Chemistry (IUPAC) nomenclature (https://iupac.org/) was also considered as it is the universally recognized authority on chemical nomenclature and terminology.

Other terms and synonyms were also collected directly from the selected use cases with the help of domain experts and literature surveys. The ontology development process relied on human feedback and decisions using a human-centered method as detailed in ref. ^46^. Finally, we integrate these concepts into SKOS hierarchies by specializing in the PO2 core model taken as a SKOS concept scheme (https://www.w3.org/TR/skos-reference/#schemes). Each SKOS concept represents a class of ontology.

### Ontology implementation and evaluation

The aim of the ontology implementation activity is to build the ontology using a formal language, based on the ontological requirements identified by the domain experts and the ontology development team.

The formalization and conceptualization of the ontology were carried out by the ontology development team, stating the concepts and relations using diagrams.net (https://www.diagrams.net/) and the Chowlk visual notation (https://chowlk.linkeddata.es/notation.html). Then, a computable model in OWL2 was generated from the ontology diagram with Chowlk Converter (see Fig. 1). The ontology code resultant from this activity include metadata, such as creator, title, publisher, license, and version of the ontology.

For the evaluation of the ontology, the natural-language competency questions collected in the specification phase were transformed into queries that were executed across the ontology. Publication and maintenance of the domain ontology are managed by specific tools as detailed in the next section.

### An ecosystem of tools developed for ontology publication and maintenance and data stewardship

**Fig. 10:**
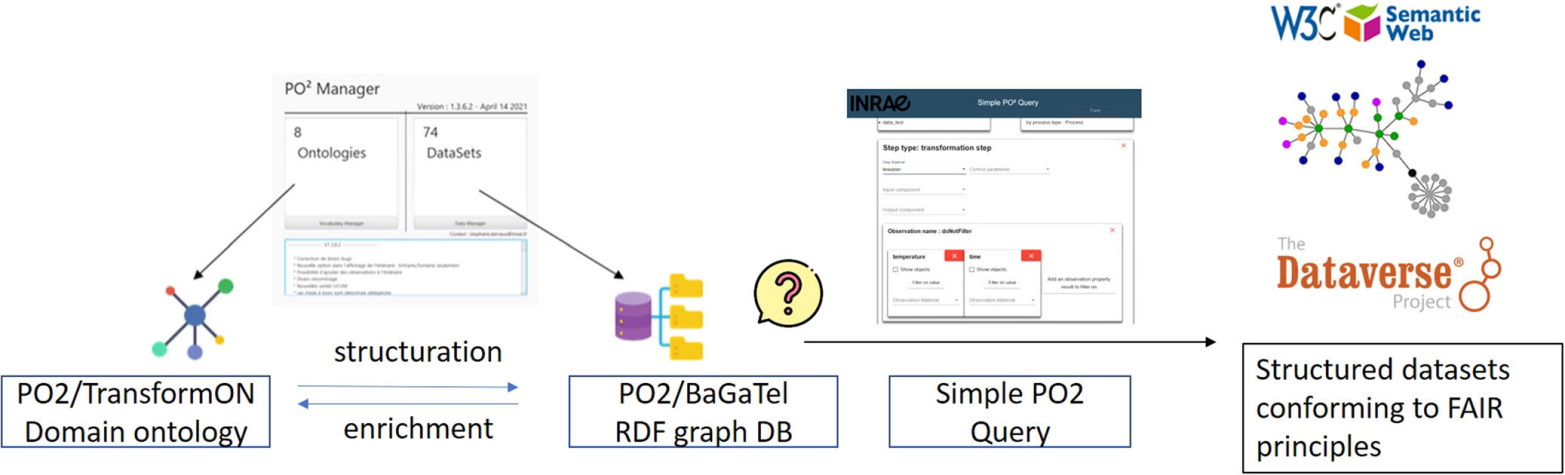
Overview of the workflow and ecosystem of tools used for ontology publication and maintenance and data stewardship. The ecosystem of tools consists in PO2 Manager, PO2/BaGaTel RDF graphDB and Simple PO2 Query tool(SPO2Q). The workflow begins with the entry of ontology-annotated data using PO2 Manager tool and ends with the deposit of structured datasets in a data repository (from left to right).

An ecosystem of tools has been designed and implemented in accordance with the PO2 core model to create and manage domain ontologies and to annotate the data which are then published into a triple store (i.e., a graph database in RDF format). Figure 10 shows the ecosystem of tools developed for ontology publication and maintenance and data stewardship.

The first tool, PO2 Manager, is a standalone application developed in Java specifically designed to assist domain experts in vocabulary management and data annotation^47^. The editor offers a graphical interface that allows a user to create processes and itineraries composed of steps (or unit operations) and input/output compositions. Observations are linked to a feature of interest (either a process, step, or composition) through this interface, and are collected either by manual entries or thanks to files in CSV format structured with specific templates. Once the dataset has been collected, PO2 Manager generates the RDF triples, and the data are published into a graph-oriented database, PO2 BaGaTel. PO2 Manager also allows domain experts to create and edit the vocabulary and publish the domain ontology with appropriate versioning into the triple store.

Another tool named “simple PO2 Query” (SPO2Q) is a web service developed to facilitate data querying from the PO2 BaGaTel RDF graph database. User forms allow end-users who do not know SPARQL, the semantic web query language, to make queries easily, by selecting appropriate fields. In an advanced usage mode, complex SPARQL queries may be defined. The queries allow data retrieval from different projects, and to reassemble data into new datasets. The datasets can then be saved or exported as CSV or JSON files. The queries can be saved and replayed when new data are available. Both queries and datasets can be published in open-access data repositories to make them publicly available.

The PO2 Manager tool and SPOQ web service are publicly available at https://quantum.mia-ps.inrae.fr/PO2/ and https://quantum.mia-ps.inrae.fr/spoq/form, respectively. The core ontology is available at 10.15454/XSVVBW with an Etalab Open License 2.0 in the open data repository Research Data Gouv. The domain ontology PO2/TransformON and its versions are available with an Etalab Open License 2.0 at 10.57745/DWX7W6 in the open data repository Research Data Gouv.

### Reporting summary

Further information on research design is available in the Nature Research Reporting Summary linked to this article.

### Supplementary information

reporting summary

## Data Availability

The PO2 core model and PO2/TransformON have been made publicly available in the repository Recherche Data Gouv, https://recherche.data.gouv.fr (accessed on 26 May 2023). The PO2 core model is available at 10.15454/XSVVBW with an Etalab Open License 2.0, compatible CC-BY 2.0. PO2/TransformON domain ontology is available at 10.57745/DWX7W6 with an Etalab Open License 2.0, compatible CC-BY 2.0.
